# The Influence of Markets on the Nutrition Transition of Hunter-Gatherers: Lessons from the Western Amazon

**DOI:** 10.3390/ijerph17176307

**Published:** 2020-08-30

**Authors:** Isabella Donders, Carles Barriocanal

**Affiliations:** 1 Institute of Environmental Science and Technology (ICTA), Autonomous University of Barcelona (UAB), Building Z, Campus UAB, 08193 Bellaterra, Barcelona, Spain; carles.barriocanal@ub.edu; 2 Department of Geography, University of Barcelona (UB), Montalegre 6, 08001 Barcelona, Spain

**Keywords:** Amazon, hunter-gatherers, indigenous peoples, market involvement, nutrition transition

## Abstract

For many centuries, hunter-gatherer societies relied on subsistence practices and traditional diets. However, forces of globalization have increased market involvement, thereby fueling the nutrition transition of hunter-gatherer societies. We review the academic literature on market involvement of hunter-gatherer societies in the Western Amazon and its consequences on diet, health and well-being. First, we elaborate on four main determinants of market involvement (accessibility, monetary income, wild meat trade and social capital), showing how each determinant draws individuals toward or away from markets. Thereafter, we discuss how these determinants alter diet, health and well-being. Our results add to the understanding of the complex relations between market involvement, dietary change, health and well-being of indigenous societies. Furthermore, they bring to light that additional research is needed on the topic to support decision-makers and help preserve indigenous values.

## 1. Introduction

The traditional diets of hunter-gatherer societies are found to be diverse and nutritious [1,2]. In addition, foods and diets provided by food systems are closely linked to health and well-being [3]. While obesity and cardiovascular diseases are major threats in industrialized countries, the lowest level of coronary artery disease ever reported is among a Latin-American hunter-gatherer society [4]. However, food systems of indigenous societies are increasingly affected by the forces of globalization [5]. An increased intake in calorically dense foods (e.g., refined sugars, saturated fats) is typical for the nutritional transition and leads to obesity and nutrition-related noncommunicable diseases (NR-NCDs), such as cardiovascular disease [6]. In addition to physical health, the erosion of social capital or identity loss are other examples of how the nutrition transition can affect the health or well-being of hunter-gatherer societies [7,8].

Increased accessibility to a physical marketplace is an important change in the food environment of hunter-gatherers. It impacts dietary choice by improving access to purchased foods including fats and sweets, while reducing access to nutritionally important foods from traditional sources [9]. Accessibility to market towns has increased due to, among other things, roads built by oil companies in hunter-gatherer territory [10,11]. Wage labor opportunities near indigenous societies can now provide people with monetary income. Improved accessibility to market towns has generated a marketplace to sell forest resources and can serve as an alternative source of income [9,11]. These shifts in livelihoods have led to the rapid change of the diets of hunter-gatherers.

The nutrition transition and its consequences are a growing area of research. This review article aims to summarize, structure and evaluate published findings about the nutrition transition of hunter-gatherer societies due to markets and its consequences on dietary diversity, health and well-being. This is achieved by first providing an overview of the main determinants of market involvement. Studying the determinants will provide the reader with an understanding of how different factors pull individuals toward or away from markets. Thereafter, we draw conclusions on how markets influence diets and fuel the nutrition transition of hunter-gatherer societies. Finally, we discuss the consequences of the nutrition transition on health and well-being. The shift away from traditional foods and its relation to risks such as chronic diseases and loss of cultural identity raises concern among researchers, policy makers and indigenous peoples themselves [8]. A limited amount of review articles has been published on dietary change or well-being of indigenous peoples in relation to market economies [12,13]. To our knowledge, no literature review has been published with the current scope, in which we focus in particular on the physical marketplace in relation to the nutrition transition of hunter-gatherer societies.

## 2. Methodology

### 2.1. Research Design, Definitions and Scoping

In this narrative literature review, existing literature on the topic has been studied. The paper does not answer one predetermined research question in particular, but is written around a central topic. A narrative literature review is, in particular, useful to summarize, structure and evaluate published findings around one central theme. We have brought together and re-structured relevant information from peer-reviewed, published literature in order to provide the reader with an overview of the state-of-the art on the topic. In addition, we have taken the opportunity to identify research gaps and propose recommendations for future research.

Data collection techniques used by researchers vary and can be both quantitative and qualitative and generally include field work carried out over multiple months or years. On-site (semi-)structured interviews and panel groups are commonly used to obtain data, complemented by observational data, questionnaires, diaries and self-reported data. Most studies rely on cross-sectional data or multivariate analysis.

Hunter-gatherer societies are located in remote areas, which makes field work resource intensive. Self-reporting and recall-techniques can reduce the reliability of data. Another limiting factor when comparing data is that contextual factors can greatly differ between societies and countries. Besides, seasonal changes can be missed depending on the duration and timing of field-work. Findings at the household- or individual level can differ from findings at the village-level and therefore results should be treated with care.

In the existing literature, multiple terms have been used to describe the occurrence and intensity of transactions between buyers and sellers at a marketplace (e.g., market involvement, market participation, market integration). The lack of agreement regarding the definition of such terms and the use of terminology makes the comparison of results challenging. Furthermore, different definitions might lead to contradictory results [14]. In this article, we chose to use the term ‘market involvement’ to describe such transactions. The word ‘market’ refers to a local and physical marketplace where goods can be purchased or sold, and therefore does not refer to a virtual marketplace, a social institution or an economic system. When we refer to studies in which other terminology was used, we only used results that were limited to a local and physical marketplace and therefore fit our scope.

**Figure 1:**
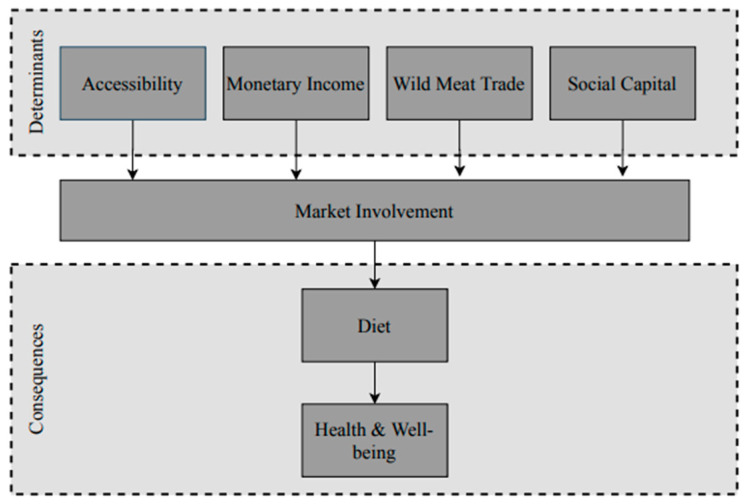
Scope of this article: determinants and consequences of market involvement.

Digital databases, such as the Scopus database, were used to obtain relevant literature. Specific combinations of keywords were used to carry out the searches. These keywords were as follows: Amazon AND [indigenous peoples OR hunter-gatherers] AND [market OR diet OR food OR nutrition transition]. These searches provided us with a wide range of English-language academic literature. A subset was selected based on relevance, year of publication and geographical area of the studied societies. Our main results were extracted from 24 articles. All articles in the subset have been published between 1999 and 2019 and study indigenous societies in the Western Amazon. Thereafter, all determinants of market involvement mentioned in the subset were collected and grouped. For example, the group ‘accessibility’ includes all articles mentioning distance, time or cost needed to reach the market. In the next step, all articles mentioning factors that can be categorized as indirect determinants of market involvement were excluded. However, some of those are mentioned in Section 6.3, in which we propose an extension of the scope of the current article. Only the groups consisting of >3 articles were kept. This method resulted in four main groups, which are referred to in this article as the ‘main determinants of market involvement’. The second part of this review studies the consequences of market involvement. Only consequences of market involvement on diet and diet-related health or well-being are part of our scope. An illustration of the scope of this review is shown in Figure 1.

### 2.2. Case Studies

**Figure 2:**
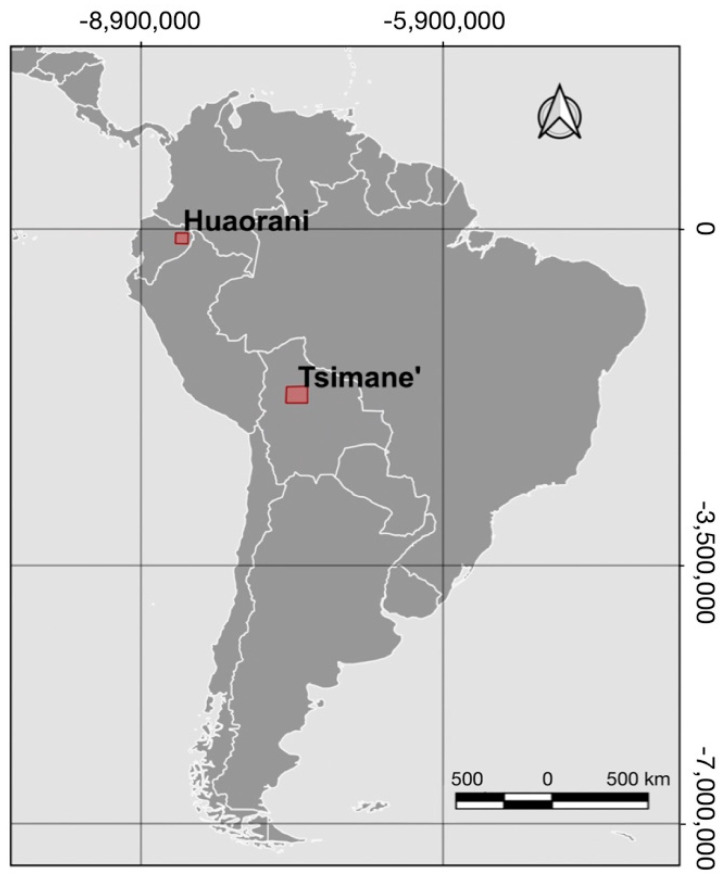
Territory of the Tsimane’ (Bolivia) and the Huaorani (Ecuador).

We focus on the nutrition transition of contemporary hunter-gatherer societies. These are societies that were hunter-gatherers in the recent past, but currently also engage in other economic activities, such as cultivation or market transactions, to cover and supplement subsistence needs [15]. We limit the review to the geographical area of the Western Amazon, with the main focus on two societies: the Tsimane’ (Bolivia) and the Huaorani (Ecuador). Both societies can be categorized as contemporary hunter-gatherers in the initial stage of market integration. There are multiple similarities between the two societies. For example, hunting is a central activity and an essential part of their culture [16,17]. Both societies consist of multiple communities living in different villages. All villages have access to one central market, but the travel time to the market differs from village to village [11,18]. Other factors, such as accessibility to wage labor or involvement in meat trade, differ between societies [9,16]. We have decided to focus our review on these two societies because both are located in the Western Amazon basin with similar natural environments (e.g., terra firme forest, soil from geological young, fluvial sediments [16,19]), which limits contextual factors, and because of the amount of published information available. An indication of the geographical location of both societies is shown in Figure 2.

#### 2.2.1. The Tsimane’

The Tsimane’ are a small-scale indigenous society in the Bolivian Amazon numbering approximately 12,000 individuals [9]. They live in around 125 permanent communities, which are settled next to roads or rivers [20]. The Tsimane’ stayed mostly isolated until the mid-twentieth century. This changed rapidly due to the arrival of Protestant and Catholic missionaries, cattle ranchers, the opening of new roads, the arrival of highland colonist farmers and the logging boom. This resulted in damage to their hunting and fishing grounds and transformation of their land tenure system and economic activities [16,21,22,23]. In addition, this led to changes in the socio-economic organization of the Tsimane’. Some Tsimane’ decided to move to more remote areas in order to maintain their traditional livelihood, while others chose to settle in permanent villages, thereby increasing their dependence on market-oriented economic activities. These changes also affected their traditions, norms and values, such as the acceptance of mononuclear families and Christian ceremonies. Over the last two decades, the Tsimane’ have been increasingly exposed to the national society as a result of the effort made by the Bolivian government. This resulted in, for example, a Spanish school curriculum, non-local teachers and the construction of basic service infrastructures. Some Tsimane’ continue to live in small villages without schools, limited contact with outsiders and only speaking the Tsimane’ language. Others live in larger villages up to 50 households with schools and speak the Spanish language [16]. Currently, for people living in those villages, wage labor opportunities include the engagement in logging camps, cattle ranches and in the homesteads of colonist farmers. Another source of income is the commercialization of forest products [9]. The market of San Borja is currently the main center for commercial transactions [24]. In addition to the currently accessible market foods, agriculture and hunting provide the Tsimane’ with their basic food needs. Agricultural activities include farming of cassava, plantains, maize, rice and chickens, for which they use slash-and-burn techniques. In addition, they gather wild fruits and hunt game and fish, which are available throughout the year [9]. There is no sale of wild game and therefore hunting is a non-cash-generating activity in the area [16,22,25].

#### 2.2.2. The Huaorani

The Huaorani (also known as Waorani) number around 2000 individuals and their territory covers the entire Yasuní Biosphere reserve, located in the Western Ecuadorian Amazon [10,26]. This area covers 16,820 km^2^ and consists of the Yasuní National Park and the Waorani Ethnic Reserve. The Huaorani were isolated, even from other indigenous groups, for a long time. The first peaceful contact was with an evangelical missionary in 1958. Two main roads were built in Huaorani territory. The first one being the Auca road, constructed in the early 1980s to facilitate oil exploration. The Huaorani were a constant threat for both missionaries and oil workers and were involved in multiple killings, including internal revenge killings. Crude oil is one of Ecuador’s main export products. Below the Yasuní Biosphere reserve lie big reserves of crude oil. This makes the area particularly attractive for oil exploration. The arrival of missionaries and oil companies had a great impact on the lives of the Huaorani, resulting in a more sedentary and missionary-dependent lifestyle, as well as epidemics such as the deadly polio epidemic of 1969 [10]. In the 1990s, the Maxus road was constructed, named after the oil company that built it. This road extends over 140 km inside the Yasuní National Park and Waorani Ethnic Reserve [10]. Hunting is an essential part of their culture and wild meat an important source of protein [17]. The Maxus Road is extensively used by hunters, thereby drastically increasing the available hunting area. Such developments continued to impact their economic, social and cultural structures. One such change is their increased market involvement. The main market is located in Pompeya, just outside the Yasuní national park, which exclusively operates on Saturdays [11]. One important factor that boasted their market involvement is the free transportation provided by the oil company along the Maxus Road, used by communities living along that road to reach the Pompeya market. Due to increased accessibility to the market, the increase in the available hunting area and improved hunting technology, the Huaorani now use a significant amount of wild meat for trade [17]. In addition, the oil company offers wage labor opportunities, which result in monetary income [26]. The aforementioned factors have significantly impacted, and continue to impact, the livelihood of the Huaorani.

## 3. Determinants of Market Involvement

Certain factors can either pull people towards or away from markets. We refer to these factors as ‘determinants of market involvement. The four determinants included in this review are: accessibility, monetary income, wild meat trade and social capital. In the following sections, we elaborate on the relation between each determinant and market involvement.

### 3.1. Accessibility

The distance between a village and the market town is widely used as a proxy for market involvement. Even though this is a relevant determinant, we should note that its limitation is that people can choose how far they live from markets [12]. Both the Tsimane’ and the Huaorani live in different villages with more or less ready access to a market.

In addition to distance, it is important to consider the time and cost needed to reach a market when studying this determinant. This can be illustrated by the difference in the number of market visits between Huaorani communities. The Dicaro community is farthest from the market (roughly 100 km). The oil company has provided them with a van and driving lessons so that they are able to travel to the market by themselves, which takes an average of 3.5 h. Guiyero is closest to the market with a distance of 32 km, but since they rely on others for transport, the travel time is less stable. They reach the market in an average of two hours, which includes up to one hour of waiting for transport. This shows that the distance to markets is not necessarily linearly related to travel time, and therefore, it is valuable to consider it separately. Besides, travel is subsidized by the oil company and therefore not a constraint. Accessibility is positively related to market involvement: people coming from Guiyero visited the Pompeya market 64% of all Saturdays during which the market was held, while Dicaro residents visited only 44% of the markets [26].

The Tsimane’ also live in different villages with more or less ready access to a central market. We do not have specific data on how these differences in distance to the market town influence the travel time or number of market visits. However, similar to the observation for the Huaorani, using the physical distance to the market as a determinant for market access is becoming less accurate due to the introduction of new ways of transport such as motorized boats [27].

### 3.2. Monetary Income

What food is selected and purchased can be dependent on income. The amount of time and personal energy available to harvest and prepare traditional cultural food items is impacted for indigenous peoples that are undergoing the transition to a wage-based economy [13].

Monetary earnings are obtained through the sale of goods and by engaging in wage labor [28]. For the Tsimane’, selling goods leads to around 56% of monetary income and wages to the other 44%. We infer that monetary income is around US$1.02 a day (our own adaptation from [28]). Gurven et al. [7] found slightly different results: that wages contributed to a bigger part of monetary income (60%) than selling goods (40%). The average monthly household income was equal to US$33 (2010 equivalent), which we convert to US$1.08 a day. Despite the participation in the market economy, the Tsimane’ still retain a high degree of economic self-sufficiency. Around 75% of adults did not report any monetary income from wage labor in a two-week time frame. In addition, just over half of the participants reported no monetary income from the sale of farm or forest goods over the same period. However, when increasing the time frame to the entire study period of 5 years, most Tsimane’ (84% of households) actually reported to have received monetary earnings [25]. Most of the money is used to acquire market goods. It was found that the total amount of cash income was significantly associated with the total weekly expenditures in market goods. Cash is not only used to purchase edible products, but also for durable commercial items, such as watches, radios and backpacks. Tsimane’ value these products as markers of status [28].

The Huaorani have the option to work for the oil company, which provides an equal daily pay rate for all individuals [17]. At the time of the study of Espinosa et al. [17], most Huaorani worked there for 3 to 4 h during the morning for 2 to 5 days a week. Lu [29] stated that in 2001, the Huaorani only spent 1% or less of household days in wage labor, and just over 10 years later, 6.9% was reported [30]. Regardless of this increase, both studies report way less involvement in wage labor than Espinosa et al. [17]. The average total income per household is around $8 dollar per day (our own adaptation from [26]). Another factor that boosted the Huaorani’s cash economy was the economic compensations provided by the oil companies to the local communities as a pay-off for the use of their territories. This led to the growth of commercial activities in Pompeya and wild meat trade became an easy way to generate additional income [11].

### 3.3. Wild Meat Trade

We only focus on the Huaorani to study wild meat trade. Even though the Tsimane’ can obtain monetary income from a variety of activities, they are not involved in the sale of game [16,22,25].

Hunting is a legal activity for the Huaorani. The price of wild meat in neighboring cities is significantly higher than the price of domestic meat. This indicates that there is a good market for wild meat. Even though hunting is legal, the trading of wild meat is illegal in Ecuador. Different institutions are monitoring wild meat trade outside national parks, such as the Ministry of Environment and Ecuador’s Environmental Police Unit. However, due to a lack of resources and the absence of law enforcement, researchers observed that market transactions can occur freely. As long as there is a great market demand for wild meat, it is likely that trade will secretly continue [11,17].

Huaorani hunters can sell meat at the Pompeya market. This market is mostly a transit point for dealers to obtain wild meat, which they can sell in other towns [11]. Houck et al. [30] state that the sale of meat is even a central source of income, with Huaorani participating in meat trade on 6.4% of household days. Suárez et al. [11] studied the wild meat transactions at the Pompeya market. In total, 47 different species were recorded at the market. The mammal species most frequently sold were the white-lipped peccary (*Tayassu pecari*), the paca (*Caniculus paca*), the collared peccary (*Pecari tajacu*) and the woolly monkey (*Lagothrix poeppiggi*). Together they represent 80% of all mammals sold. Espinosa et al. [17] found as well that the white-lipped peccary and collared peccary were mostly traded (75% of all biomass traded). For settlements close to markets and focusing on the percentage of harvested individuals, the woolly monkey and paca came next. However, when studying the total weight of biomass traded, they did not stand out that clearly. Two other species that did stand out in this regard (after the white-lipped peccary and collared peccary) were the South American tapir and the red brocket. Since Espinosa et al. [17] did not only study meat trade, but all harvest, they could estimate that around 35% of all meat harvested was traded. Remarkably, the meat prices were very homogeneous across species as well as across seasons, and independent of the total amount of meat sold [11,17,26]. The only exception seems to be the paca, which was consistently sold at a higher price, and which also remained higher along the entire market chain. Pacas were sold for an average price of US$3.34 ± 0.74/kg, versus US$2.11 ± 0.52/kg for all other meat [11]. This latter price was confirmed by the study of Franzen and Eaves [26]. The study of Espinosa et al. [17] confirmed that the only exception to the homogeneous pricing is the paca, with the reason being its preferred taste.

Contradictory evidence is found regarding which factor incentivizes hunters to sell more meat. While Franzen and Eaves [26] found that more meat is sold when the hunter harvests more per hour, from Sierra et al. [31] it seems that to sell more meat, hunters will allocate more time hunting. On the contrary, when there are more wage labor opportunities, hunters will likely reduce time hunting, due to increased opportunity cost [26,31,32]. Meat trade became a more attractive activity for the Huaorani communities along the Maxus road, since the transportation cost of those communities was greatly reduced by the transportation provided by the oil company. Suárez et al. [11] stated that the Huaorani hunters of these villages account for one third of all biomass traded and that if the transportation subsidies were absent, the expectation is that they would sell virtually no meat at the market. This is based on the significant correlation found between the actual transportation costs between communities and the Pompeya market, and the amount of biomass of wild meat sold (r^2^ = 0.51; *p* = 0.015). In addition, it seems that the closer a household is to a market, the more animals are hunted [31].

### 3.4. Social Capital

Markets can erode social capital if one chooses to sell surplus on the market and thereby decrease participation in kin-based exchange networks. Selling surplus on the market can be used as a risk-mitigation strategy since storable products can be purchased in return. Such products have the advantage of being both storable and fungible: they can be saved and used during a period of food shortage and they are easily exchanged for different resources of similar value [7]. Sharing carries risk: others may not reciprocate as expected. When market goods are used as a risk-mitigation strategy, it can influence diets if traditional foods are partly replaced by storable (market) foods. While some studies show that market access indeed weakens reciprocity [33], Henrich et al. [34] state that societies whose diets consist of more market foods show more generous social behavior and share more. In addition, maintaining a reputation for sharing is important for other reasons, such as receiving support from kin during times of injury or illness [35].

Gurven et al. [7] found that greater market involvement did not substantially displace sharing networks of the Tsimane’. It is suggested that the reason for this is that greater market involvement does not appear to buffer households against experiencing idiosyncratic shocks or facilitate recovery [36]. However, the latter study also shows that, even though the Tsimane’ clearly practice intensive food sharing and reciprocity, only 5% received help from kin after unforeseen income shocks, such as crop loss. In addition, Tsimane’ closer to markets show other individualistic behavior, such as building walls around their homes, putting fences around their courtyards or putting locks on their doors when leaving to the village town [36,37].

Franzen and Eaves [26] reached a similar conclusion for the Huaorani. They noticed that the amount of surplus sold at the market has no relation with sharing intensity. Their results suggest that selling surplus (meat in this case) has lower expected benefits than sharing. This means that sharing requirements will first be satisfied before excess meat will be sold on the market.

## 4. Influence of Markets on Diet

Both societies still obtain the largest part of their foods from the natural environment: horticultural field products, fish and wild game. However, market foods such as processed carbohydrates, nonhunted meat, sweets and condiments are currently also frequently encountered. In the next two sections, we describe how market foods have re-shaped the diets of the Tsimane’ and the Huaorani. Thereafter, we elaborate on the relation between each determinant of market involvement and dietary change of both societies.

### 4.1. Dietary Change of the Tsimane’

The Tsimane’ diet is characterized by a high proportion of carbohydrates and low amounts of fat. The most recent data show that the largest part of the (non-market) dietary energy comes from cultivated staples (61.9%), followed by fish (15.6%), meat from domestic animals (7.5%) and wild game (6.1%) [27]. Older estimates are similar, with the biggest difference being that the dietary energy coming from fish and wild game seems to be interchanged [38]. Other estimates state that the meat comes from game (0.48 kg/person/day), fish (0.31 kg/person/day) and beef from livestock (0.25 kg/person/day) [39]. The diet of Tsimane’ children differs from the diet of adults: the dietary diversity seems higher and children consume more fruits and eggs, but less flesh meat than adults [40].

The consumption of market foods is on the rise, with 61% of households reporting market purchases over a one week study period [41]. In particular, the amount of refined sugar, salt and oil consumption has increased significantly over the last years. Sugar and oil consumption have increased by 15.8 g/day and 4.9 mL/day, respectively [27]. This change was reported between 2010 and 2015, but before 2010, the sugar and oil intakes were already increasing, albeit less rapidly [42]. The total dietary energy intake of market goods is estimated to be between 2% and 8% of the total dietary energy intake, with the highest percentage being the most recent [27,36,38]. The main market foods encountered are pasta, flour, bread, sugar, oil and meat [27,28].

### 4.2. Dietary Change of the Huaorani

The Huaorani are still very dependent on the forest and spend most of their days (63.7% of household-days) exploiting wild foods [29]. Wild meat is the most important source of protein. Field work from 2000 suggests that wild meat is present in 74% of meals [26]. The amount of animal species hunted varies between 41 and 72 and seems to be fairly stable over the last three decades [31,43]. The Huaorani consume an approximate amount of meat between 0.24 and 0.32 kg/person/day [17,44].

Purchased foods are frequently consumed as well. In the interviews, 82% of the households mentioned that they consume purchased staples (e.g., rice), and 34% that they consume canned protein (e.g., tuna) [26]. However, Lu [29] mentions that a market transaction takes place only during 2.2% of household-days. This suggests that when a market transaction takes place, large amounts of foods are purchased, or that the evidence from these studies is contradictory. It can also be due to the limited amount of study samples on Saturdays, which is the only day that the market is operative.

### 4.3. Accessibility

Reyes et al. [9] concluded that villages further away from markets have greater dietary diversity than villages close to markets. Drawing upon the (adapted) guidelines of the FAO Guidelines for Assessing Dietary Diversity [45], they grouped all food consumed by Tsimane’ households in 12 food groups. They found that the dietary diversity of Tsimane’ living in remote villages was about 0.5 food groups higher than Tsimane’ living closer to the market (*p* < 0.001). The higher dietary diversity was due to the fact that nutritionally important food groups were consumed more frequently. This is in line with the expectations of researchers working at the end of the 20th century, who predicted that the dietary diversity of indigenous peoples will decrease if they rely on more limited market foods and use less of their traditionally harvested food [13,46]. In the remote villages, products such as meat, fish, legumes, nuts, milk and milk products were consumed more frequently. Foods that were most often purchased at the market were from the food groups ‘milk and milk products’ (100% of these foods were obtained from the market), ‘sweets’ and ‘spices, condiments & beverages’. For these food groups, no significant difference between villages close and far from markets could be found. This difference was more significant in the food group ‘oils & fats’, since the remote villages got 83.5% of the foods in this group from the market, versus only 37.6% for the closer villages. Furthermore, intakes of total energy and carbohydrates increased significantly over the years, where the rate of increase was higher for villages closer to market towns. However, total net energy and macronutrient intakes are still greater in remote villages. This can be explained by the fact that the population densities are higher closer to markets, which leads to poorer access to hunted and fished food, or due to sporadic incomes, which lead to decreased food availability [27].

Espinosa et al. [17] show that, while studying the Huaorani, the amount of meat kept for self-consumption (and therefore not used for trade) did not differ between communities living close or far from markets. Evidence suggests that market foods are more often encountered in the diets of Huaorani with greater market accessibility [47]. Recent studies show that the LOSS (lard, oil, sugar, salt) acquisition is not correlated with distance to market towns [27], whereas previous studies suggested otherwise [37]. As mentioned earlier, this might be because new transportation options reduce the importance of physical distance to market towns.

### 4.4. Monetary Income

Reyes et al. [9] compared different profiles related to time allocation (foragers, agriculturalists, wage labor, diversifiers) and found no clear difference in dietary diversity. The source of food groups was not compared to time allocation in that study, but Lu et al. [29] studied this relation during their work on five different societies in Ecuador. They reported a positive correlation between wage labor, and the frequency of purchasing food and the consumption of nonhunted meat. More specific data is needed to show the exact increase in purchased goods in relation monetary income.

Rosinger et al. [41] show that for the Tsimane’, purchase behavior differs per expenditure category. They divided the sample over expenditure groups depending on the amount of money spent on (food and non-food) items. While, for example, expenditures on processed carbohydrate increased steadily over expenditure categories, purchased meat was common across all categories, despite it being relatively expensive. Godoy et al. [25] found no statistically significant association between monetary income and wildlife consumption. This is in line with the finding that the amount of meat kept for self-consumption does not change with the presence of markets, which will be explained in the next section.

### 4.5. Wild Meat Trade

The amount of meat consumed by the Huaorani only slightly increased over the last 30 years [17,43]. This shows that even with the current exposure to a market, meat trade and more advanced hunting technology, the amount of meat consumed has not significantly changed. Based on these findings, we therefore expect that only surplus is sold on the market. This is in line with Sierra et al. [31], who explain that the amount of food produced by communities that rely on subsistence practices is based on maintaining a culturally defined level of comfort. If more is produced, surplus resources are used to improve that minimum acceptable level of comfort, and are often used for risk-mitigation. One of these risk-mitigation strategies can be selling surplus meat on the market in order to obtain cash, which can be used to buy storable foods in return. However, to corroborate these statements, more specific data on the direct relation between purchasing market foods and wild meat trade is needed, and is, to our knowledge, not available to date.

### 4.6. Social Capital

Goods entering a Tsimane’ household as gifts accounted for 6.7% of the total value of household consumption. The proportions of households that gave away edible goods during a one-week time-frame were as follows: 71% of households shared home brewed beer, 58% shared cooked food, 45% shared plantains, 42% shared meat, followed by rice (37%), fish (32%), manioc (31%) and maize (28%) [36].

The Huaorani practice intensive sharing as well. Franzen and Eaves [26] state that Huaorani households report to receive wild meat from other households in 48% of the interviews, as well as garden foods in 31%. However, they report to receive purchased foods in only 8% of the interviews, while they report consuming it in 82%. Therefore, purchased foods are the most likely to remain in the household, whereas foods that are shared are obtained from the wild.

## 5. Influence of Markets on Health and Well-Being

Although money can be used to obtain food or other necessary goods at the market, it does not always contribute to better health. Apart from necessities, money is used to buy food that is high in carbohydrates, sweets, alcohol and cigarettes [7,28,37]. Popkin [6] projected the current situation accurately in 2004, when he stated that diets in the developing world are changing rapidly and that the increased intake in calorically dense foods is typical for the nutrition transition and leads to obesity and NR-NCDs. In the next section, we elaborate on the dual burden of malnutrition and its relation to the intensity of market involvement. Thereafter, we concentrate on the relation between health and the main determinants of market involvement. The last section is dedicated to the relation between markets and subjective well-being.

### 5.1. The Dual Burden of Malnutrition

The dual burden of malnutrition is the paradoxical phenomenon of the coexistence between overweight and underweight individuals. This has been observed within households, but also within larger groups, such as villages or societies [30,48]. In addition, children that were once underweight, may become overweight once market foods become accessible. Malnourished or underweight infants, who undergo such a shift and become overweight, also have an increased risk of NR-NCDs [2,6]. Houck et al. [30] show the presence of the dual burden of malnutrition in Ecuador. While studying seven different indigenous communities, they found that high rates of stunting were present, indicating chronic malnutrition. They included two Huaorani communities in this study: the Gareno and Quehuereono communities. Remarkably, the Gareno has one of the highest rates of overweight (11–16%), while overweight was not present in the Quehuereono. In addition, the Gareno community was more actively involved in wage labor and meat trade than the Quehuereono community, and had easier access to roads and markets. This suggests that monetary income and market access can lead to over-nutrition, and at the same time, can co-exist with high rates of stunting. Two other communities included in the same study were the Kichwa community and the Shuar community. An interesting finding was the high rates of stunting (44–60%) among the Kichwa community in relation to their great involvement in market activities. It seems that not only the net amount of market involvement is important, since the Shuar community engage in a comparable amount of market activities while showing lower stunting rates. One explanation is that the Shuar have a longer and more stable involvement in market activities, while, for the Kichwa, these activities increased over the last 10 years. This would mean that a rapid increase in market involvement might be more impactful than the actual net involvement.

In both Bolivia and Ecuador, food insecurity and chronic malnutrition has been an issue for many years. Average children’s stunting rates are above 50% for indigenous peoples in both countries and the ethnic divide is striking. Stunting rates of indigenous peoples are twice as high compared to the rest of the society [49]. In addition, there is a positive relation between obesity prevalence and higher GDP levels. This association is weaker in females than males [50]. However, obesity seems to more common in women than in men [41]. Wells et al. [50] suggest that this difference is largely connected to national wealth inequality and gender inequality. These findings highlight the importance of improving women’s status in order to address the global obesity epidemic. These findings are valid on a macro-level, but do not seem to consider the importance of local dynamics such as rapid economic changes. In addition to the findings of Wells et al. [50], Rosigner et al. [41] state that in such situations, changes in body composition, such as the likelihood of being overweight and an increase in BMI, are related to market expenditures. They show stronger results for men, due to the fact that mostly men are participating in local markets, which increases the access to market foods [41,51]. Women’s activity levels related to food production are therefore not reduced in the same way as men’s when the levels of market involvement increase.

### 5.2. Determinants of Market Involvement

In this section, we elaborate on the relation between two main determinants of market involvement and health. We only focus on the determinants ‘accessibility’ and ‘monetary income’. We do not include the determinant ‘wild meat trade’ due to the lack of information available about the relation between this determinant and purchasing market foods, as stated in the previous chapter. The fourth determinant, human capital, is excluded since we have shown before that markets do not necessarily erode social capital, and therefore, we will not be able to show a strong relation between social capital, market involvement and health. However, there are a limited number of published findings of other societies in the Western Amazon that show that reciprocity in-kind stopped functioning due to the appearance of markets. For example, the Shipibo (Peru) stopped sharing meat when they decided to focus on selling rice [33]. We elaborate on this lack of data, and provide recommendations for future research, in the next chapter.

Godoy et al. [36] studied the covariates of nutritional status among Tsimane’ adults in the early stage of participation in the market economy. From all the measured village-related variables, only the ‘village-to-town walking time’ (which can be compared to ‘accessibility’) correlated strongly and reliably with anthropometric indices of nutritional status. Such anthropometric indices of nutritional status are often used as objective measures to assess health when other more invasive methods (e.g., collecting blood samples) are rejected by the societies or not available [12]. The positive correlations found are between village-to-town walking time and age and sex-standardized z-scores of mid-arm muscle area and body-mass index. Mid-arm muscle area is widely used as a general measure for under nutrition and obesity risk. Mid-arm muscle area is an index of muscle development and protein reserves. Village-to-town walking time was negatively correlated with the age and sex-standardized z-scores of skinfolds, which are a measure of energy reserves as body fatness [36]. We infer from this that people in villages further away from markets have a lower risk of obesity or protein-deficiency, whereas people in villages closer to a market town tend to have more body fat. Byron [37] demonstrates a relation between self-perceived illness of children and proximity to the market town: the closer to the market, the greater the duration and severity of illness. In addition, alcohol use and abuse cause problems for the Tsimane’. Extreme drinking behavior is more common and problematic closer to the market town. In addition, the frequency of smoking is significantly higher closer to the market town (*p* < 0.001) [37].

Researchers have looked for correlations between these same indices for nutritional status and monetary income. Results suggest that monetary income obtained from wage labor does not have a statistically significant effect on anthropogenic indices of short-run nutritional status [52]. Rosinger et al. [41] showed a small increase in BMI, weight and percentage of body fat when market food expenditures increase. This was not a statistically significant result, however. Gurven et al. [7] found that higher wage income is related to less illnesses or accidents, whereas Byron [37] did not find a relation between economic and social integration into the market economy and health among Tsimane’ adults. The discrepancy might occur because Byron [37] compared health to more variables than only wage income. Interestingly, household heads with higher incomes from wage labor are also twice as likely to have a sick spouse. It might, however, be possible that a sick spouse is actually the motivation to engage in wage labor [7].

### 5.3. Subjective Well-Being

Some community-members indicate concerns about the increased consumption of purchased goods, as well as the relation to identity loss [8]. The Kichwa of Ecuador, for example, mention being concerned about chronic diseases such as diabetes in conjunction with the dietary transition. As a result of the popularity of processed foods, children’s food preferences have changed: Kichwa children commonly reject home-cooked traditional meals, as they prefer to eat sweets, candy and other market foods. In addition, the Kichwa are concerned about how they lose food as a means to maintain their traditional ancestral heritage [8]. Another example is supplied by Tsimane’ women, who report concerns about their husbands who spend wage earnings on alcohol instead of necessities [37]. Masferrer-Dodas et al. [28] state, however, that consumption of market goods is not associated with the subjective well-being of the Tsimane’. They argue that this might be due to the fact that the sense of well-being of the Tsimane’ is more centered around social relations and success in subsistence activities than around market-related activities. Whether the dietary transition does indeed influence one’s well-being seems to be a subjective matter and culturally determined.

## 6. Discussion and Recommendations for Future Research

In the next section we briefly discuss the main findings per determinant, as well as identify research gaps. After this, we show the importance of proper management of factors related to market functioning (e.g., wild meat trade, road construction) in order to preserve healthy ecosystems. In the last section, we propose an extension of the current scope for future research, in order to augment and deepen the current knowledge on the relation between markets, diet and health.

### 6.1. Determinants of Market Involvement

Increased accessibility to market towns seems to increase market involvement. To quantify the accessibility, the physical distance as well as the time and cost to reach the market should be considered, as we have observed that physical distance is not always linearly related to time or cost. Villages close to market towns are more subject to changes in their consumption and show a lower dietary diversity. Alcohol use and abuse has a great direct impact on consumers and their direct relationships.

Both the Tsimane’ and Huaorani still retain a high degree of economic self-sufficiency, despite wage labor opportunities. It remains unclear whether wage labor and the resulting monetary income have a positive or negative effect on diet and health. Wage labor is positively correlated with purchasing market foods, but the relation with health is complex. Health consequences might become more evident as time passes and when potential consequences (such as chronic diseases) are observed or are more widely studied. Multiple factors appear to be important for a final conclusion. On the one hand, cash leads to different (increased) purchasing behavior with its related health consequences. On the other hand, cash can influence compensational factors, such as the accessibility to (non-traditional) health care, with potential health benefits.

Wild meat trade does not appear to directly influence diet, as we have shown that mostly surplus is sold on the market while self-consumption is not altered. Cash obtained from the trading of game can be spent on the market and in this way influences diet, but we do not have specific data to illustrate this pattern. For future research, we recommend the study of this particular relation in order to gain a better understanding of the importance of wild meat trade on market involvement and the resulting dietary changes and health consequences.

Markets are not a direct threat to social capital for either the Tsimane’ or the Huaorani. Therefore, it cannot be assumed that once indigenous societies become more market oriented, they lose their subsistence practices. While studying other societies in the Western Amazon, researchers have obtained results both supporting [29] and contradicting these findings [33]. We identify a need for further research to determine which factors are likely to cause misfunctioning of in-kind reciprocity. Most foods that are shared come from the natural environment, whereas market foods stay in the household and are used for risk-mitigation and diversification.

### 6.2. Implications for Conservation

The Huaorani extract a significant amount of wild meat, both for self-consumption and for trade. Animals extracted from the forest also include vulnerable species [11,26,44]. The extraction rate of some species, such the white-lipped peccary, is above sustainable levels. Depletion of these species can lead to changes in top-down ecosystem processes, due to the loss of top predators [17]. Deforestation and forest fragmentation due to cash-cropping, oil exploration, logging companies, cattle ranchers, and highland colonists in turn affect wildlife populations [17,53,54]. Changes of the environment introduced by external factors, such as roads built by oil companies, continue to impact the environment, even when resource extraction activities are terminated. Especially in tropical areas, where pressure on land and natural resources is high and environmental institutions are weak, this can result in illegal hunting, colonization and associated habitat loss, increased fragmentation and degradation [17]. Investment in policies and regulation is of great importance to avoid such destructive consequences, and when properly managed, can even increase wildlife abundance, as examples have shown [55].

### 6.3. Interrelations and Prerequisites

What becomes clear from the analysis of determinants is that it is difficult to assess them independently. We found that interrelations between determinants are present and need to be well understood in order to make conclusions about the influence of individual determinants on either market involvement, diet or health. Studying these interrelations is beyond our scope, but we identify a need for further research on the interrelations between determinants. As an example, in this review, we do not find a clear relation between the determinant ‘social capital’ and market involvement, diet or health. However, multiple studies have identified important interrelations between this determinant and others. For instance, it has been found that an increase in monetary income among the Tsimane’ may be both positively [56] and negatively [33] associated with social capital. Moreover, various relations between wild meat trade and accessibility to market towns have been published [11,17,26]. Such examples can be found in a wide range of literature. In addition to these interrelations, the prerequisites for the determinants also need to be better understood. For instance: education and Spanish fluency are shown to be important prerequisites for obtaining wage labor or earning higher wages [28,57]. In addition, education is associated in various other ways with dietary diversity and nutritional status [13,36,40,53].

We indicate a need for a future review of the interrelations between determinants, as well as the related prerequisites, thereby extending the scope of the current article. Broadening the scope will lead to a deeper understanding of the nonlinear and complex relations between markets, diet and health. This allows one to better predict the consequences of changing one or multiple determinants. We expect that this information will be valuable for decision- and policy makers and should be carefully considered when proposing changes that will impact hunter-gatherer territories.

## 7. Conclusions

Market foods have been increasingly identified in the diets of hunter-gatherer societies. Using two societies in Bolivia and Ecuador as case studies, we have elaborated on which determinants incentivize people to move toward or away from markets. Villages closer to markets have a lower dietary diversity, show a greater increase in total energy and macronutrient intake, and suffer more from adverse effects due to alcohol and smoking. Monetary income has been linked to the frequency of purchasing food, but more data is needed to show its effect on diet. The (illegal) sale of wild meat stimulates hunters to visit markets, but how this influences purchasing behavior remains to be understood. Both societies retain a high degree of economic self-sufficiency and social capital has not been eroded. Currently, markets do not draw the Tsimane’ nor the Huaorani away from subsistence practices. Rather, market goods are used for risk-mitigation and diversification. The consumption of calorically dense market foods, such as sugar and oil, is on the rise. Closer to markets, people seem to have more body fat, a higher risk of obesity, consume more alcohol and smoke more frequently. Indigenous community-members indicate concerns about the increased consumption of market foods and its relation to chronic diseases and identity loss.

This article adds to our understanding of how markets alter the diets and resulting health and well-being of contemporary hunter-gatherer societies in the Western Amazon. The relation between markets, diet and health is complex and more research is needed to fill current research gaps. A better understanding of these complex relations will be of great importance to decision- and policy makers who aim to introduce changes that will impact hunter-gatherer territory, as well as to indigenous peoples themselves to shape their decision-making processes and protect their health. With broad knowledge on the topic, the direct and indirect consequences of proposed changes can be better predicted. This knowledge can then be used, both top-down and bottom-up, to support indigenous societies, prevent adverse health effects and preserve indigenous values.
